# Supplementary material for: Cannabinoids modulate food preference and consumption in Drosophila melanogaster
Source: Sci Rep. 2021 Feb 25;11:4709. doi: 10.1038/s41598-021-84180-2 (PMC7907270; doi:10.1038/s41598-021-84180-2)
Supplement: Supplementary file 1 — Supplementary Information [file 41598_2021_84180_MOESM1_ESM.pdf]

Cannabinoids modulate food preference and consumption in *Drosophila melanogaster*

Jianzheng He[1,*], Alice Mei Xien Tan[1], Si Yun Ng[1], Menglong Rui[1], Fengwei Yu[1,2,3,*]

[1]Temasek Life Sciences Laboratory, 1 Research Link, National University of Singapore, Singapore 117604.

[2]Department of Biological Sciences, National University of Singapore, Singapore 117543.

[3]NUS Graduate School for Integrative Sciences and Engineering, Centre for Life Sciences, Singapore 117456.

***Corresponding authors: hejianzheng1006@163.com; fengwei@tll.org.sg**

Running title: Cannabinoids affect feeding behavior in flies

Supplementary

Figure S1

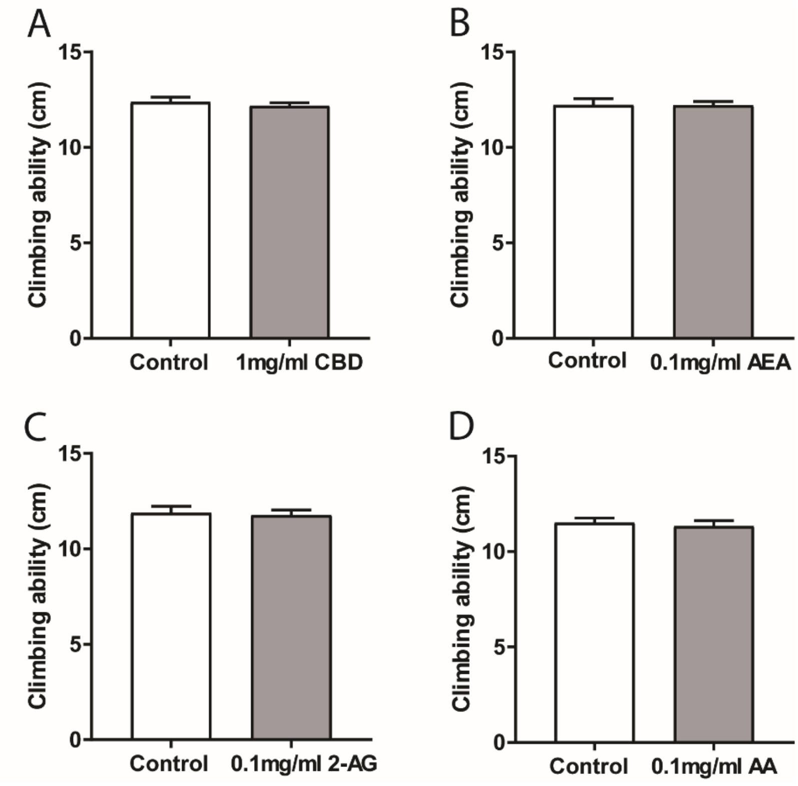

Figure S1. No or negligible effects of cannabinoids on climbing ability of the treated flies. Following two days of treatment with cannabinoids, the climbing ability of the flies was assessed to determine the locomotive behavior. Pre-treatment with 1 mg/ml CBD (**A**), 0.1 mg/ml AEA (**B**), 0.1 mg/ml 2-AG (**C**), 0.1 mg/ml AA (**D**) (n=8-12 vials/group) did not alter the climbing performance. Data are represented as mean ± S.E.M.

Figure S2

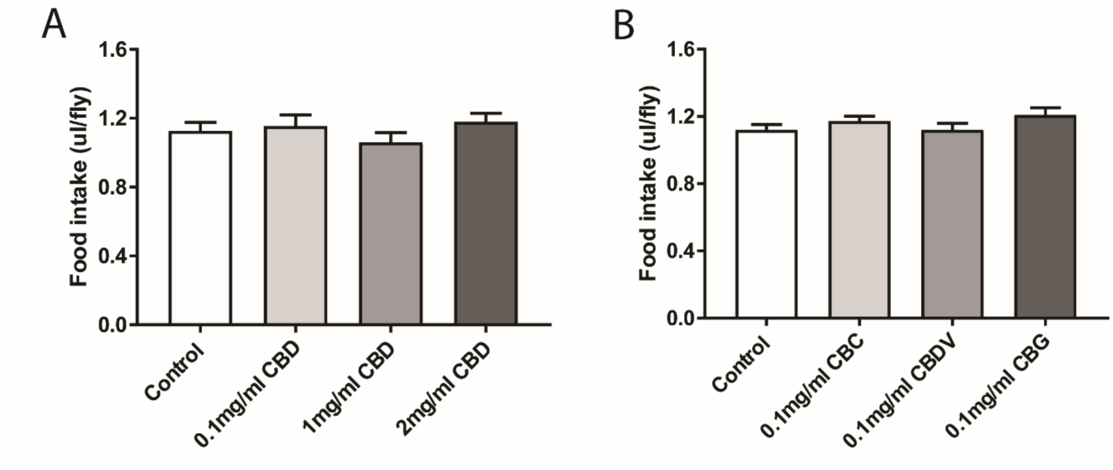

Figure S2. Initial food intake was not altered during the phytocannabinoids training. Flies consumed similar amounts of food containing various concentrations of CBD (0.01, 0.1and 1 mg/ml; n=12-19 vials/group) (**A**), or CBDV, CBC or CBG (0.1 mg/ml; n-32-33 vials/group) (**B**) during two days of the training (day -2 and day -1) as compared to the respective control solutions. Data are represented as mean ± S.E.M.

Figure S3

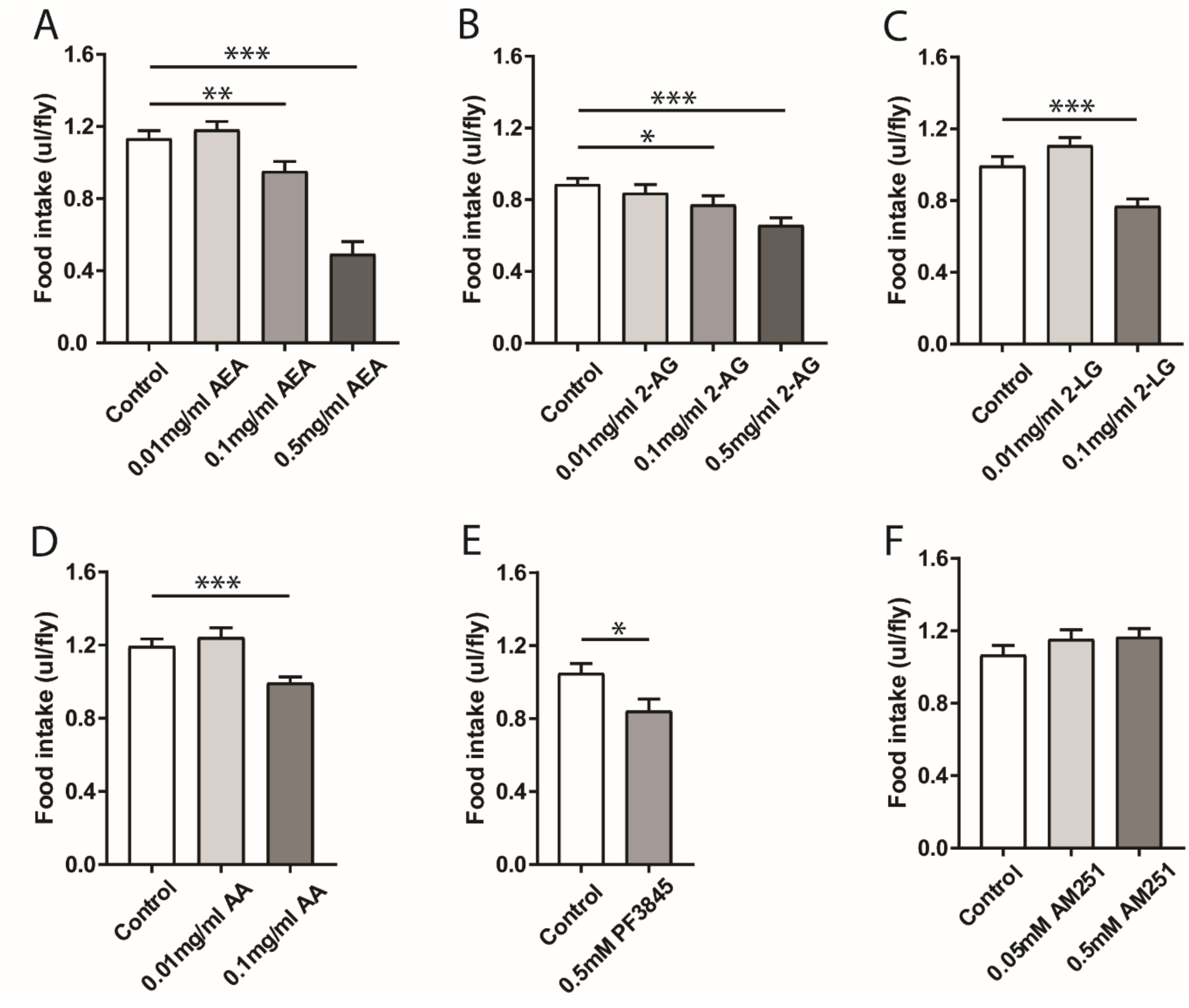

Figure S3. Endocannabinoids-containing food intake decreased during the initial training. The amount of food with AEA (0.1 and 0.5 mg/ml) (**A**), 2-AG (0.1 and 0.5 mg/ml) (**B**), 2-LG (0.1 mg/ml) (**C**), AA (0.1 mg/ml) (**D**) and PF3845 (0.5 mM) (**E**) consumed by the flies during two days of the training (day -2 and day -1) was significantly lower when compared to the respective control solutions (n=10-27). The consumption of food with AM251 (**F**) by the flies did not differ from the control groups (n=18 vials/group). Data are represented as mean ± SEM. One-way ANOVA followed by Dunnett's post-test was applied to determine statistical significance. *p<0.05, **p<0.01, and ***p<0.001.
